# Microsatellite genotyping of clinical Candida parapsilosis isolates

**DOI:** 10.29252/cmm.3.4.15

**Published:** 2017-12

**Authors:** Hamid Badali, Sassan Rezaie, Jacques. F Meis, Setareh Agha Kuchak Afshari, Mona Modiri, Ferry Hagen, Maryam Moazeni, Rasoul Mohammadi, Sadegh Khodavaisy

**Affiliations:** 1 Department of Medical Mycology/Invasive Fungi Research Center (IFRC), School of Medicine, Mazandaran University of Medical Sciences, Sari, Iran; 2 Department of Medical Mycology and Parasitology, Tehran University of Medical Sciences, Tehran, Iran; 3 Department of Medical Microbiology and Infectious Diseases, Canisius-Wilhelmina Hospital, Nijmegen, Netherlands; 4 Centre of Expertise in Mycology Radboudumc/CWZ, Department of Medical Microbiology Nijmegen, Netherlands; 5 Department of Medical Parasitology and Mycology, School of Medicine/Infectious Diseases and Tropical Medicine Research Center, Isfahan University of Medical Sciences, Isfahan, Iran

**Keywords:** *Candida parapsilosis*, Genotyping, Iran, Microsatellite

## Abstract

**Background and Purpose:**

*Candida parapsilosis* is a predominant species found in nosocomial infection, particularly in hospitalized patients. The molecular epidemiology of the clinical strains of this species has not been well studied. The present study was performed with the aim of investigating the microsatellite genotyping of *Candida parapsilosis* among the Iranian clinical isolates.

**Materials and Methods:**

This study was conducted on 81 independent clinical *C. parapsilosis* isolates that were genotyped by using a panel of six microsatellite markers.

**Results:**

The short tandem repeat (STR) typing of clinical *C. parapsilosis* isolates demonstrated 68 separate genotypes, among which 57 genotypes were observed once and the remaining 11 cases were identified for multiple times. The Simpson’s diversity index for the panel of combined six markers yielded a diversity index of 0.9951. The heterogeneity was observed among the Iranian and the Netherlands clinical *C. parapsilosis* isolates.

**Conclusion:**

As the findings indicated, the clinical *C. parapsilosis* isolates from Iran showed a high genetic diversity. It can be concluded that molecular epidemiology could be useful for screening during outbreak investigation where *C. parapsilosis* is involved.

## Introduction

The incidence of *Candida* infection associated with non-*albicans Candida* (*NAC*) species has been increasing throughout the world [1, 2]. *Candida parapsilosis* is one of the most frequent *NAC* species, which causes a broad spectrum of infections from superficial to invasive candidiasis [3, 4]. These species can lead to nosocomial infection, particularly in hospitalized neonatal and pediatric patients through catheters, intravascular devices, and hand carriage of the health care workers [5-8]. The accurate identification of *C. parapsilosis* strains is crucially important for the adoption of the appropriate treatment regarding the different antifungal susceptibility patterns of *C. parapsilosis* complex [9, 10]. *C. parapsilosis* has been reported to account for several cases of fungemia outbreak in hospitalized patients. Based on genotypic analysis, these outbreaks were probably due to cross-infections by health care workers [11]. Since the isolates of *C. parapsilosis* complex are indistinguishable based on their phenotypic features, molecular assays have been developed for distinguishing and genotyping these species [12, 13]. Among these molecular methods, microsatellite analysis has been used for molecular typing of the species belonging to *C. parapsilosis* and other fungi [14, 15]. Therefore, the current study aimed to apply microsatellite analysis for genotyping of clinical *C. parapsilosis* isolates from Iran. 

## Materials

***Isolate identiﬁcation***

A total of 81 *C. parapsilosis* clinical isolates were investigated using molecular typing method. All clinical isolates were grown for 48 h on Sabouraud glucose agar (Merck, Germany) at 30°C. Initial species identification was performed based on conventional tools using CHROMagar *Candida* medium (CHROMagar Microbiology, Paris, France) at 30°C [22]. DNA was extracted using the CinnaPure DNA isolation kit (Sinaclon, Iran)in accordance with the manufacturer’s recommendations. The DNA concentration was adjusted as approximately 25 ng/µL and stored at -20°C prior to use. The identities of *C. parapsilosis* isolates were confirmed by the partial sequencing of the internal transcribed spacer DNA region as previously described [16].

**Table 1:** Amplification primers used for microsatellite genotyping of *Candida parapsilosis* isolates

**Marker**	**Labeled primer (5`–3`)**	Unlabeled primer (5`–3`)*	**Repeat Unit**
3A	FAM-CCTGGCTTGCAATTTCATTT	GCCTCATCGGTGGTGGAATTA	TCT
3B	HEX-TTGGAGTAACAAGCGCAGAA	GTCGCTTGGACAACTGGTGTA	TTG
3C	TAMRA-CAATAGCAGCAATGGAGCAG	GTGCTTTTGGTTTGTCCTTGG	AAC
6A	FAM-CCAGGTTGGACTATCACTG	GGTTTCATTTTGTTGTGAAAA	TGCTTT
6B	HEX-CCCTTTCAAAAGAAACAGACA	GTTCTATAGATAAAACACACCCCATACA	AGTGTT
6C	TAMRA-TGGCGTTAGTATTGGCGTTA	GATTGTATCACGCGGGAACTC	TGTTGG

* The underlined G nucleotide in the unlabeled primer sequence is not a match to the genomic DNA, but was introduced to minimize the formation of minus-A peaks.

***Microsatellite typing***

A panel of six short tandem repeat (STR) markers was used for typing all clinical isolates of* C. parapsilosis*. Generally, three trinucleotide repeat markers and three hexanucleotide markers were amplified in a multiplex polymerase chain reaction (PCR). In addition, each amplification primers were labelled at the 5 side with 6-carboxy-fluorescein (6-FAM), either 6-carboxy-tetramethylrhodamine (TAMRA) or hexachloro-fluorescein (HEX). The primer sequences (Table 1) and PCR amplification reactions were followed as described before [14]. Briefly, the amplification products were prepared for analysis by 100-fold dilution with distilled water. Subsequently, 1 µL of the diluted PCR product was added to 8.75 µL ddH_2_O and 0.25 µL internal size marker ET-ROX 400 (GE Healthcare, Diegem, Belgium). In the next step, the samples were boiled at 95°C for 1 min, subjected to rapid cooling at 4°C, and then injected and run onto ABI3500xL Genetic Analyzer platform (Applied Biosystems, Foster City, CA, USA) as recommended by the manufacturer. The number of repeats in each marker was achieved by comparing the relative size of each allele with those obtained by means of the reference *C. parapsilosis* strain CDC317. The similarities between the genotypes were visualized by constructing a minimum spanning tree (MST) using BioNumerics, version 6.0 (Applied Maths, St.-Martens-Latem, Belgium). The discriminatory power was calculated by Simpson’s index of diversity as described previously [17]. The study was approved by the Ethics Committee of Tehran University of Medical Sciences, Tehran, Iran.

## Results

**Figure 1:**
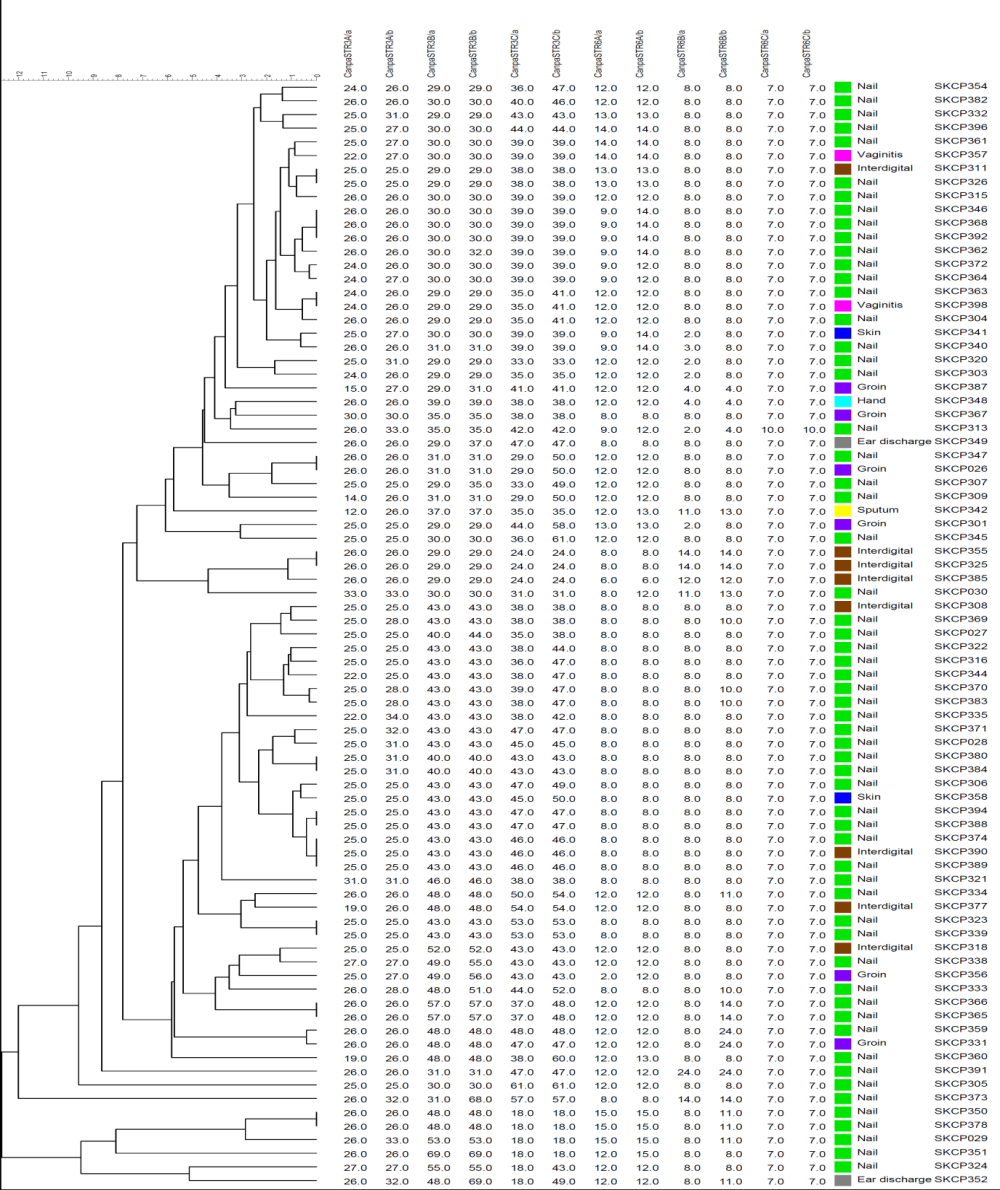
Dendrogram showing genotypic diversity among 81 clinical *C. parapsilosis* isolates obtained from Iran. The scale bar indicates the percentage similarity between the genotypes. The columns after the short tandem repeat patterns represent the source of isolates and isolate number, respectively.

**Figure 2:**
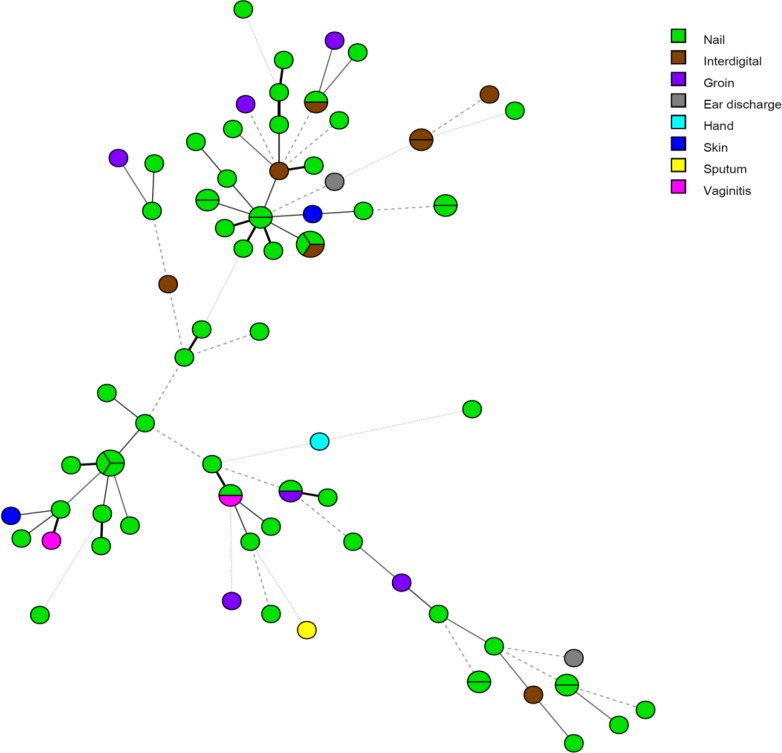
Minimum spanning tree showing the diversity between the genotypes based on sample type categorized analysis. Each circle represents a unique genotype, and the circle size is correlated with the number of isolates belonging to the same genotype.

**Figure 3:**
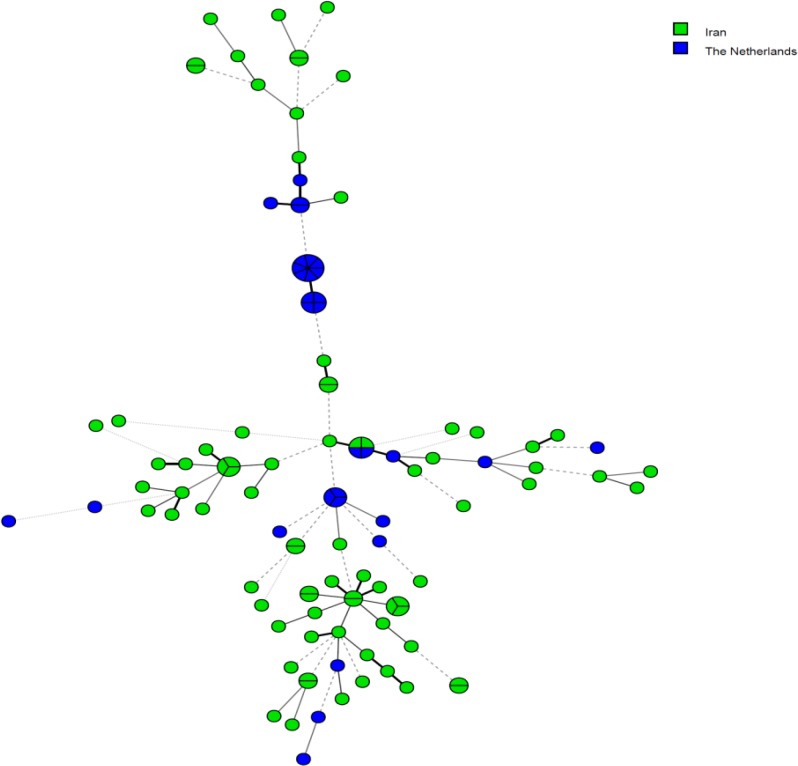
Minimum spanning tree representing the genotypic diversity based on the origin of *C. parapsilosis* isolates. The green and blue circles indicate Iranian isolates (n=68) and isolates obtained from the Netherlands (n=18), respectively.

Totally, 81 clinical *C. parapsilosis *isolates were identified from a variety of clinical specimens, including nail scraping (n=59), interdigital skin (n=8), groin (n=6), vaginal (n=2), ear discharge (n=2), skin (n=2), hand (n=1), and sputum (n=1). The STR typing of 81 *C. parapsilosis* isolates demonstrated 68 separate genotypes. The genetic relatedness of clinical *C. parapsilosis* isolates is depicted in figure 1. Out of all genotypes, 57 cases were observed once, and the remaining 11 genotypes were identified for multiple times. Nine genotype clusters were shared between two *C. parapsilosis* isolates, and also two genotypes were shared among three *C. parapsilosis* isolates. One cluster, including three isolates (i.e., SKCP346, SKCP368, and SKCP392), obtained from different patients of the nail samples, showed the same allelic profile at six loci in STR typing. In addition, one similar genotype was found to be related to three *C. parapsilosis* isolates (i.e., SKCP374, SKCP390, and SKCP389), which were obtained from various specimens of different patients. Furthermore, the same genotype was detected to be related to two *C. parapsilosis *isolates (i.e., SKCP311 and SKCP326) obtained from different anatomic sites. Moreover, three patients with candidiasis were sampled twice on two different days, and the same genotype was observed over the time. In Figure 2, MST represents the genotypic diversity of clinical *C. parapsilosis* isolates based on sample type analysis. The STR typing revealed a high genetic diversity in the Iranian *C. parapsilosis* isolates, compared to other clinical isolates from the Netherlands. Based on the MST (Figure 3), it was clear that two clinical isolates from Iran and two clinical isolates from Netherlands were in the same clonal cluster. The Simpson’s diversity index for the individual markers ranged within 0.2666-0.0933, and the panel of all six markers yielded a diversity index of 0.9951.

## Discussion

Recently, NAC species has became an increasing prevalence among the hospitalized patients. Some of these species exhibit decreased susceptibility to the commonly used antifungal agents [7, 18, 19]. This aspect has caused a concern among the clinicians about the potentiality for the emergence of antifungal resistance [18, 20]. *C. parapsilosis* is well known for nosocomial spread through hospital environment [6, 21, 22]. Moreover, this species has a worldwide distribution [23-25]. The discrimination of the *C. parapsilosis* isolates was accomplished by the development of the DNA-based typing methods [26-28]. Various molecular methods, including PCR-restriction fragment length polymorphism (RFLP), PCR-based random amplified polymorphic DNA (RAPD) technique, matrix assisted laser desorption ionization-time of flight mass spectrometry, and multilocus sequence typing, have been described for the identification of *C. parapsilosis* complex species in the previous studies [29-31]. Notably, STR typing assay has been reported to have a high reproducibility and discriminatory power. This assay is well utilized as a powerful tool for the specific identification of several yeast species [32-34]. Lasker* et al.* used a microsatellite method for the genotyping of *C. parapsilosis* isolates based on dinucleotide repeats; however, they reported some limitations in this regard [35]. In agreement with our results, Sabino *et al. *also described polymorphic microsatellite markers with higher discriminatory power (0.99) for the differentiation of *C. parapsilosis* isolates and reported this approach as a reliable method for molecular epidemiological studies [36]. They also asserted that microsatellite markers have high reproducibility and are potential to identify multiple genotypes for *C. parapsilosis* isolates. Therefore, they marked that this method has incomparable advantage over other typing methods, such as RAPD, PCR-RFLP, and internal transcribed sequence grouping [36]. This panel of six markers allow for excellent discrimination between the isolates from different origins. In the present study, we characterized clinical *C. parapsilosis* isolates by using six-marker microsatellite panel assay. As mentioned above, the majority of the clinical *C. parapsilosis* isolates had unique genotypes. In this regard, within the collection of 81 *C. parapsilosis* isolates obtained from clinical sources, 68 unique genotypes were observed. The pattern results of the STR analysis of two hexanucleotide markers revealed no variation between all *C. parapsilosis* isolates, except one isolate (SKCP313) that had diversity at these two loci, whereas high allelic variation was observed at other locus. In the present study, the isolation of the related genotypes of *C. parapsilosis* from multiple anatomical sites over time supported the evidence of an endogenous colonization. In line with our results, Diab-Elschahawi *et al.* reported 24% clonally related genotypes from multiple anatomical sites in patients [14]. In addition, similarity was observed just in a clonal cluster between the Iranian and Netherlands clinical isolates, indicating high genetic diversity of clinical *C. parapsilosis* isolates inside and outside Iran. 

## Conclusion

In conclusion, the Iranian *C. parapsilosis* isolates were found to have a high genetic diversity. Microsatellite genotyping method could be useful for screening during outbreak investigation, especially where *C. parapsilosis *complex is involved.
